# Annotations

**Published:** 1902-04-26

**Authors:** 


### Annotations.

### Deaf Children.

At the last meeting of the Otological Society Mr. Arthur H. Cheatle presented a report upon the results obtained by an examination of the ears of 1,000 children at the Hanwell Schools, results which may well give rise to anxiety, whether we regard them from a medical, an educational, or a sociological point of view. The object which he had in view in making this investigation was in the first instance to ascertain the amount of ear disease in children of the poorest class, and what he found was sufficiently startling, for of the 1,000 children examined more than half failed to pass the test for normal hearing. Many of these, especially those with depressed membranes associated with adenoids, had not been noticed by their teachers to be deaf, the deficiency only being discovered on examination. It is little less than appalling to hear that of the 1,000 children examined no less than 335 were, or had been, affected with discharge from one or both ears. The association of adenoids with deafness comes out very strikingly in this report, for 394 of the 434 children in whom they were present showed also some aural troubles. That something ought to be done to prevent the continuance of this shocking state of affairs goes without saying. What that something ought to be is another question. The answer of some will doubtless take the direction of the systematic inspection of school children by skilled medical inspectors and, as an almost necessary corollary, the establishment in connection with the elementary schools of an otological department. But the problem goes far deeper than that. No one can doubt that the prevalence of adenoids and suppurative diseases of the ear, is largely due to conditions of life, to the dirt, overcrowding, lack of air, lack of light, and ill-considered, if not absolutely deficient, diet, which are the prominent characteristics of the home life of too many of the children of the poor, or that in any serious attempt to stem this torrent of deafness, which threatens to dull the intellectual vivacity of the race, something much more radical is required than a mere tinkering of diseased ears.

### Exploiting a Prescription.

There are many ways of dealing with a prescription. One may throw it away after having the medicine made up, or one may preserve it, in deference to the direction "bring this prescription with you" sometimes printed upon it; or, as some faddy people do, one may paste it in a book with all sorts of notes appended as to the condition for which it was given, and as to its efficacy or the opposite; or one may lend it to such of our acquaintances as appear to the uninstructed eye to be suffering "just in the same way"; one may even so lend it "for a consideration," and so get some return for one's own sufferings and the doctor's fees. It has been reserved, however, for a man in Liverpool to discover a still better way. According to the evidence given in a recent trial, the said Liverpudlian had a wife who suffered from an affection of the throat, for the treatment of which he obtained some pastilles. Afterwards, at his request, the medical man from whom they were obtained wrote out for him a prescription for some less costly lozenges, and armed with this, he seems to have gone to a large firm of wholesale druggists and arranged that they should manufacture and sell these lozenges, paying a royalty of 6d. per lb. That the defendant was at the time secretary of the doctor's private hospital, that the druggists maintained that according to their understanding of the arrangement the royalty was intended to go to the hospital, and that no such royalty had been received by that institution, were the leading facts on which the druggists sought to set aside the agreement, and on which the court gave judgment in their favour; but all that has nothing to do with the point to which we wish to draw attention, namely, that a person having obtained from a medical practitioner a prescription for a useful sort of lozenge, was able to plant it like a little seed upon that fertile soil the British consumer of proprietary medicines, and then sit still and watch it grow and multiply, until in the course of a few years the royalties came pouring in at the rate of over £800 a year!

### The Weaker Vessel.

The usual cry of women workers is for fair play, they undersell men with very little conscience. But the trades in which men and women work under exactly equal conditions are so few that it is not always easy to say whether or not it is fair that women's pay should be less than men's or whether their work is as good as their male competitors'. In the cigar industry, however, it seems that women are on exactly the same footing as men. A lady who has investigated the conditions of this industry, Mrs. Oakeshoot, states that in this trade there is no opposition to women on the part of men; the women belong to the same union, work in the same shops, and have a foothold in factories where the best work is done; and since each individual in a factory works entirely by himself, there is nothing to prevent a woman who shows herself capable of it from doing the same work as men. But they don't do it. "They are slower and on the whole less skilful than men; they lack incentive, they like to 'lark a bit,' and they are addicted to marriage." As men are addicted to marriage also, it may seem strange that this should be set down as reason for the comparative failure of the woman worker. But the man who is married or means to marry looks at his work differently from a woman with similar views. He knows that by his work he must maintain his wife and family as long as he lives. But the woman who thinks of marrying has in her mind the notion that soon her husband will take her away from the factory for ever. Perhaps she is wrong. All women don't marry, and all married women do not get free from the necessity of working for their living. But that is not how the average factory girl or other woman worker looks at the matter, and the result is that in the cigar industry "the maximum of the women's earnings is found to be the minimum of the men's, and they are content with an average of 15 shillings a week." Doubtless these differences of inclination and habit obtain in other trades, and possibly they explain why, as a rule, women are paid less than men; but it is not often that circumstances show the industrial inferiority of women so clearly as this trade of cigar-making.

